# Contrast Enhanced Micro-Computed Tomography Resolves the 3-Dimensional Morphology of the Cardiac Conduction System in Mammalian Hearts

**DOI:** 10.1371/journal.pone.0035299

**Published:** 2012-04-11

**Authors:** Robert S. Stephenson, Mark R. Boyett, George Hart, Theodora Nikolaidou, Xue Cai, Antonio F. Corno, Nelson Alphonso, Nathan Jeffery, Jonathan C. Jarvis

**Affiliations:** 1 Department of Musculoskeletal Biology, Institute of Ageing & Chronic Disease, University of Liverpool, Liverpool, Merseyside, United Kingdom; 2 Cardiovascular Research Group, School of Medicine, University of Manchester, Manchester, Greater Manchester, United Kingdom; 3 Prince Salman Heart Center, King Fahad Medical City, Riyadh, Kingdom of Saudi Arabia; 4 Alder Hey Children’s NHS Foundation Trust, Liverpool, Merseyside, United Kingdom; University of Notre Dame, United States of America

## Abstract

The general anatomy of the cardiac conduction system (CCS) has been known for 100 years, but its complex and irregular three-dimensional (3D) geometry is not so well understood. This is largely because the conducting tissue is not distinct from the surrounding tissue by dissection. The best descriptions of its anatomy come from studies based on serial sectioning of samples taken from the appropriate areas of the heart. Low X-ray attenuation has formerly ruled out micro-computed tomography (micro-CT) as a modality to resolve internal structures of soft tissue, but incorporation of iodine, which has a high molecular weight, into those tissues enhances the differential attenuation of X-rays and allows visualisation of fine detail in embryos and skeletal muscle. Here, with the use of a iodine based contrast agent (I_2_KI), we present contrast enhanced micro-CT images of cardiac tissue from rat and rabbit in which the three major subdivisions of the CCS can be differentiated from the surrounding contractile myocardium and visualised in 3D. Structures identified include the sinoatrial node (SAN) and the atrioventricular conduction axis: the penetrating bundle, His bundle, the bundle branches and the Purkinje network. Although the current findings are consistent with existing anatomical representations, the representations shown here offer superior resolution and are the first 3D representations of the CCS within a single intact mammalian heart.

## Introduction

The cardiac conduction system (CCS) is a collection of specialised tissues within the heart responsible for the initiation and coordination of the heart beat: the CCS initiates the cardiac action potential and conducts it throughout the heart [1]. The three major components are the sinoatrial node (SAN), the atrioventricular node (AVN) and the His-Purkinje system. The functioning of the CCS is dependent on the geometry of the CCS as well as its electrical properties. Our present understanding of the geometry of the sinoatrial and atrioventricular nodes owes much to painstaking reconstruction of three-dimensional (3D) anatomies from the two-dimensional (2D) section of cardiac tissue [2], [3], [4], [5] In such reconstructions, boundaries must be drawn in each section between areas of conducting tissue and the surrounding contractile myocardium. 2D areas representing the CCS must then be reconstructed as ‘Z-stacks’ that represent the 3D volume. The distinction between conducting tissue and the surrounding myocardium or connective tissue can be based on morphological differences between populations of cells, or differential staining techniques such as Masson’s trichrome [3], [4] or Periodic Acid Schiff (PAS) staining [6], [7]. Alternatively, staining techniques that bear a direct relationship to the function of the CCS can be used, for example immunohistochemistry with antibodies directed against specific ion channels that are present at high concentration in the membrane of conducting myocytes [8]. However, any such technique is limited in resolution to the distance between successive sections, typically 60–340 µm [3], [4]. Any improvement in resolution is at the expense of a proportional increase in the number of sections that must be individually processed and analysed. Furthermore, such destructive analysis is often limited by the requirement to make a preparation of an isolated part of the heart, small enough for routine fixation and sectioning. In this case, there is another source of error in placing the segment back into a model of the whole heart. In any technique in which successive sections are physically separated, there is the problem of accurate registration between one section and the next in the reconstructed image. In episcopic microscopy images are taken of an immobilised sample as sections are progressively removed. This deals with the problem of registration, because the relationship between the sample and the image is fixed, but brings its own restrictions. In general, because of the difficulty of staining intact specimens, the contrast between areas of interest of the sample is based on autofluorescence or negative staining. In principle, an episcopic technique could be used based on a fluorescent marker that selectively stains the CCS in a whole heart [9], but to our knowledge this has not yet been achieved. 3D reconstructions of the sinoatrial and atrioventricular nodes of the rabbit heart have been generated based on high resolution (∼25 µm) overnight magnetic resonance imaging (MRI) [10]. MRI has also been used to generate a 3D reconstruction of the major free running Purkinje fibres in the rabbit heart [11]. However, the major free running Purkinje fibres constitute only a small fraction of the Purkinje network. The entire Purkinje network of the rabbit heart has been stained and photographed, but this resulted in a 2D representation only of the Purkinje network [12]. MRI has the advantage of providing high resolution non-invasive imaging of 3D morphology. However, it has the disadvantage of long acquisition times and suffers from practical limitations (spatial resolution, signal:noise, cost) as previously discussed [13]. Recently, high resolution micro-computed tomography (micro-CT) imaging using iodine as a contrast agent has been introduced for *in vivo* [14], [15], [16], [17] and *ex vivo* [13], [18], [19], [20], [21] anatomical studies. We have applied the technique to the imaging of cardiac structures, and focus in this paper is on the CCS. Micro-CT imaging has the advantages of MRI, but has the additional advantages of fast acquisition times (minutes) and spatial resolutions almost an order of magnitude higher.

In the current study we have observed the major components of the CCS in the hearts of rats and rabbits, in such a way that the normal anatomical relationships are preserved in the micro-CT images. The images represent as nearly as possible the geometry that pertains during normal cardiac function. Image analysis of the scanned data has allowed us to present the first 3D representation of the three major components of the CCS within a single dataset, along with its *in situ* relationship with the cardiac mass.

## Materials and Methods

### Ethics Statement

The animals from which samples were obtained were in a study reviewed by the UK Home Office Inspectorate under the Animals (Scientific Procedures) Act in the UK. Project Licence 40/3135 was granted to authorise the work. As part of the review process, the Animal Welfare Committee of the University of Liverpool also scrutinised and approved the experimental work. All sampling procedures were performed under deep anaesthesia or after death.

### Rat Hearts

Micro-CT contrast enhancement is dependent on the use of contrast agents, optimised for concentration, incubation time and specimen size [13]. To seek a practical technique to prepare cardiac tissue for CT imaging, hearts were dissected from 4 adult rats and fixed in phosphate buffered formal saline (PBFS - formaldehyde dissolved as a 4% solution in phosphate buffered saline) for at least 48 hours. This allows storage and fixation with limited tissue shrinkage. The PBFS solution was changed as necessary to avoid acidic conditions. The samples were then stained in iodine potassium iodide (I_2_KI) in aqueous solution (I_2_KI at 1.87, 3.75, 7.5 or 15%) for 2 days (see table 1).

**Table 1:** Sample preparation and scanning conditions.

Sample	I_2_KI (%)	Incubation (days)	kV/µA	Isotropic voxel size (mm)	PBFS bleeding (weeks)
***Rattus Domesticus***					
Rat Heart 1	15	2	145/120	0.0197	4
Rat Heart 2	7.5	2	135/110	0.0185	3
Rat Heart 3	3.75	2	130/115	0.0132	2
Rat Heart 4	1.87	2	140/120	0.0186	2
***O. cuniculus***					
Rabbit Heart 1	7.5	3	155/150	0.0402	5
Rabbit Heart 2	3.75	5	145/140	0.0423	5
Rabbit Heart 3	3.75	5	135/120	0.0444	5
Rabbit Failure	7.5	3	155/140	0.042	5

Showing, the concentration and incubation time used in the staining process. The x-ray conditions used and resolution obtained for the scanning process (Isotropic voxel size represents 50% of the obtained resolutions, as raw data was reconstruction at 50%, see methods). The longevity of bleeding required to extract residing contrast agent from the tissue.

### Rabbit Hearts

4 Adult rabbit hearts were prepared for imaging. 3 were from control animals and 1 from an animal after the induction of experimental heart failure (aortic valve destruction followed by aortic banding). Systemic heparin was administered prior to termination by intravenous injection under isoflurane aneshesia (2000 Units/kg). The rabbits were then terminated by overdose of sodium pentobarbitone. The thoracic cavity was exposed and the inferior vena cava and descending aorta cannulated. In order to clear the heart of blood the chambers were flushed *in situ* via the inferior vena cava with heparinised saline (5000 Units/ml) until the expelled solution ran clear. The heart was then fixed by a period of perfusion with PBFS by retrograde injection via the aorta. In specimens in which the aortic valve was intact, blanching of the coronary arteries was achieved with no further intervention. In samples from rabbits whose aortic valve was damaged to create an experimental model of left heart failure, it was necessary to divert the flow into the coronary arteries by pressure applied to the ventricles, and sometimes to the head and neck vessels. Samples were then fixed by a period of perfusion with PBFS (approx 10 minutes) via the aorta and, if necessary, via the venae cavae to naturally inflate all of the heart chambers with PBFS. The partially fixed hearts were then removed to separate vials containing PBFS for further long-term fixation. The inflated state of the hearts was maintained by gentle agitation until all chambers were full of fixative and the hearts were floating freely without contact with the vial walls; particular care was taken over the thin-walled atria. They were then stained in iodine potassium iodide (I_2_KI) in aqueous solution (I_2_KI at 3.75 or 7.5) for 3–5 days (see table 1).

### Imaging

Each specimen was removed from the staining solution, rinsed with PBFS to remove excess stain and to prevent surface saturation, drained and blotted. The spaces within the heart and great vessels were thus filled only with air. The samples were then triple-bagged (in compliance with local regulations for the use of the CT scanner) and securely supported centrally in a plastic (radiotranslucent) tube by placing additional thin polythene bags. This allowed us to align the specimens with the axes of the micro-CT scanner and to reduce the likelihood of rotational artefacts in the slice reconstructions. All specimens were micro-CT imaged with the Metris X-Tec custom 320 kV bay system at the EPSRC-funded Henry Moseley X-Ray imaging Facility, Manchester University. Imaging parameters were optimised for each specimen to maximise spatial and contrast resolution and to facilitate data handling. Scans were acquired using a copper target with x-ray energies ranging from 130–155 kV. 1440–3500 projections within the 360° rotation were recorded, resulting in scan times of approximately 20–50 minutes. All scans were reconstructed at 50% of the obtained resolution to aid post processing and analysis, isotropic resolutions ranged from 13–44 µm (details given in table 1).

### Histological Verification of CT Identification

Initial observations of the enhanced micro-CT data suggested that the conduction system could be identified by differential attenuation. To confirm this, the contrast agent was leached out of the scanned rat hearts by replacing the fixative with fresh PBFS at weekly intervals until yellow colouration in the fixative surrounding the sample ceased. The samples were then embedded in parraffin wax, sectioned on a microtome at 5 µm and stained with Masson’s Trichrome for light microscopy. The CCS was indentified both by its lighter staining in comparison to the surrounding myocardium, and its association with known anatomical landmarks, including the leaflets of the aortic and tricuspid valves. ImageJ 1.45i (http://rsbweb.nih.gov/ij/) was used to virtually re-slice the isotropic micro-CT data for each heart to find a 2D plane which matched the morphological appearance, and the associated landmarks identified in each stained histological section. This is only possible because micro-CT imaging produces true 3-D datasets with isometric voxels, which can be re-sliced accurately in any plane.

### Image Handling

Data sets were viewed, manipulated and analysed using ImageJ 1.45i (http://rsbweb.nih.gov/ij/). Regions of interest were sought initially using known landmarks, as used in histological studies of the CCS, including the crista terminalis and the intercaval region for identification of the SAN, and the membranous interventricular septum (IVS) and cusps of the aortic valve for the AV conduction axis. Once identified, structures were reconstructed in 3D using volume rendering (VRT) and semi-automatic segmentation techniques in Amira 5.33 (see below). The resultant 3D meshes made up of the voxels attributed to the CCS components could be manipulated and cropped in any orientation and their respective volumes, surface areas and dimensions quantified.

### Semi-automatic Segmentation

In this approach, pixel values representing X-ray attenuation within the anatomically defined CCS are recorded. A masking window is then created that identifies further structures of the CCS, on the basis that they will have similar pixel values, and thus discriminates the CCS from the surrounding tissue. The ‘magic wand’ tool is then used at the defined window settings to segment the structure slice by slice, that is to identify contiguous blocks of pixels that correspond to the defined window of attenuation levels. The ‘magic wand’ tool implements a recursive seed fill method of filling 2D graphic images; the selected seed is allowed to grow selecting adjacent pixels under the constraint of the masking window. The efficiency of the technique can be improved by the use of the interpolation function which anticipates the position of a structure over multiple slices between two reference planes.

Where there was an overlap of pixel values between the CCS and the surrounding tissues the limit line function was implemented, to make an objective identification of pixels near to boundaries between one level of attenuation and another, and to reduce noise in the resultant isosurface.

### Volume Rendering

In this automatic technique, an opacity curve is windowed to include voxel values of interest and a 3D colour map selected. The resultant 3D volume is rendered using a VRT render mode in which voxels are assigned a level of opacity (0–100%) calculated from their CT attenuation value.

### Superposition of Segmented Images with Ghosted (pseudocolour) CT Image

In this approach, two separate identically scaled isosurfaces are created from the same data set; one encompassing the entire cardiac mass created using the threshold segmentation function, and the other representing a subset of the dataset- in this case the semi-automatically segmented CCS. The result is two correctly aligned isosurfaces. The transparency of the overlying cardiac mass is then adjusted to reveal the *in situ* position of the CCS.

## Results

**Figure 1:**
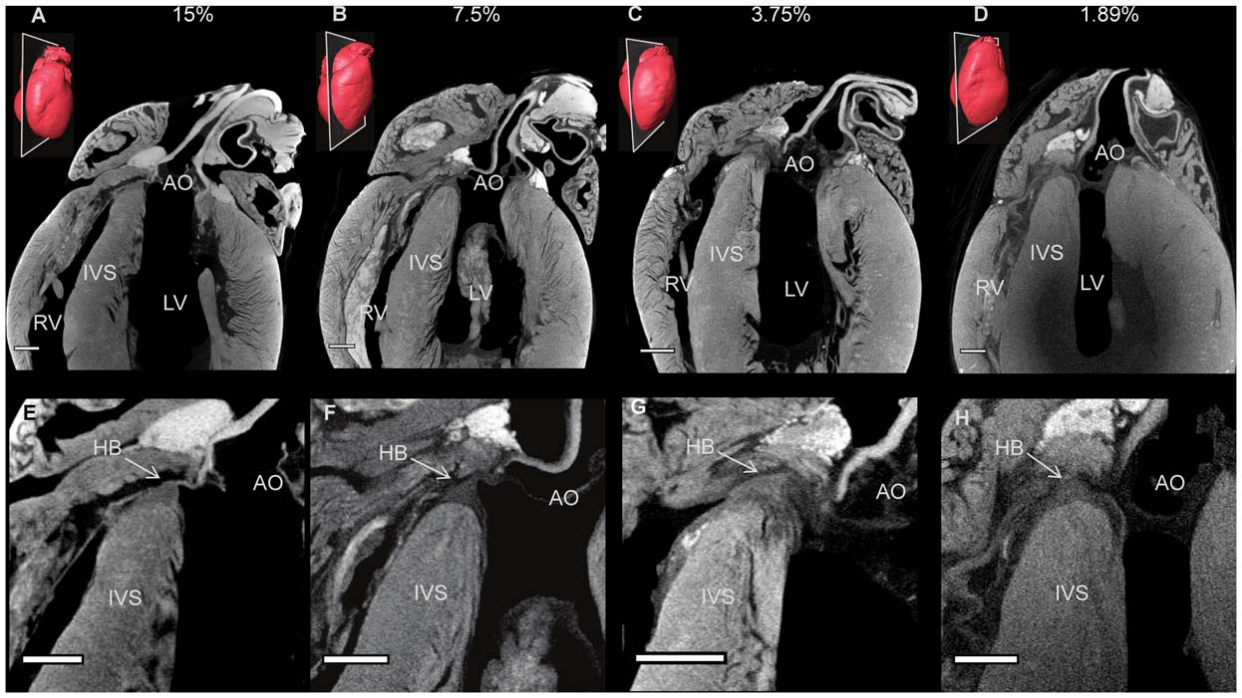
Optimization of the micro-CT imaging technique. Longitudinal micro-CT images (A-D), through 4 rat hearts stained with 15, 7.5, 3.75 and 1.87% concentrations of I_2_KI respectively. Corresponding high power images focussing on the atrioventricular conduction axis (E-H). Inset 3D renderings of each heart show the associated planes of interest. AO- aorta, IVS- inter-ventricular septum, HB- His bundle, LV- left ventricle, RV- right ventricle. (scale bar represents 1 mm).

**Figure 2:**
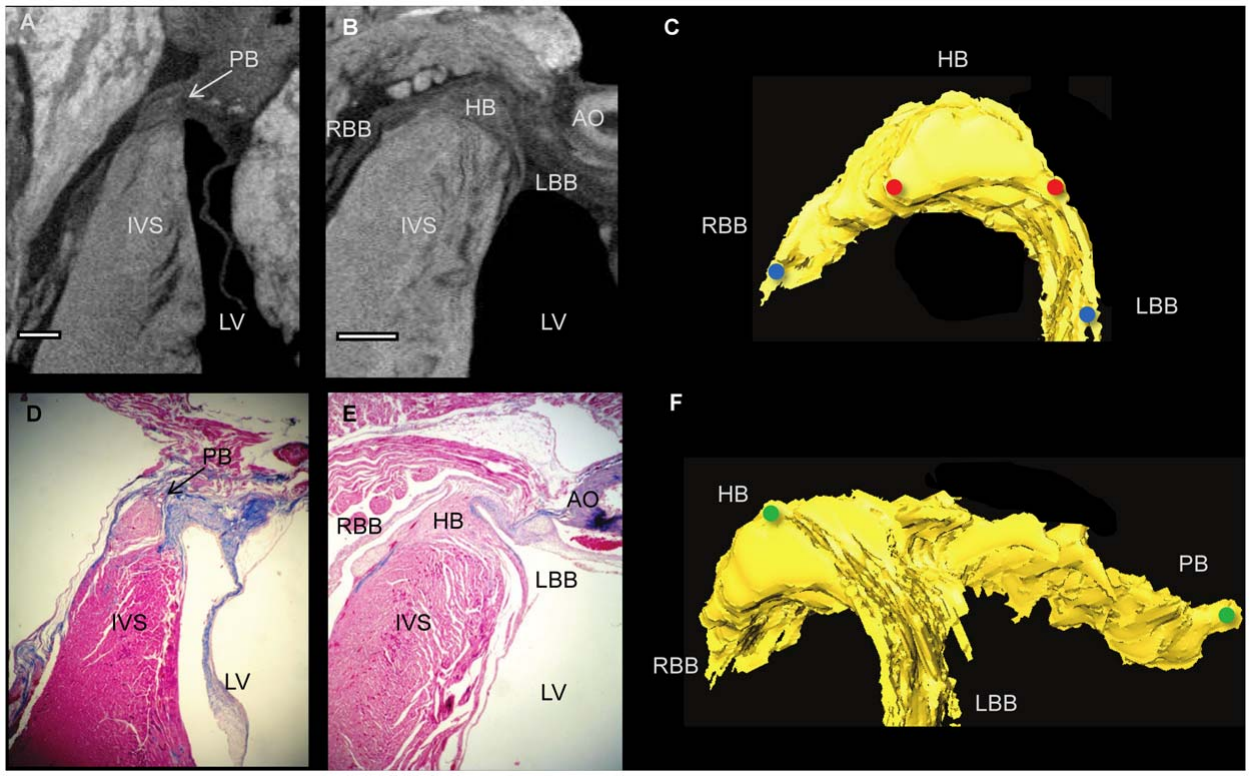
The atrioventricular conduction axis in rat. Longitudinal micro-CT images showing the penetrating bundle and His bundle in rat hearts stained with 7.5% (A) and 3.75% (B) I_2_KI for 2 days. Corresponding histological sections stained with Masson Trichrome (D,E). Segmented 3D surfaces of atrioventricular conduction axis, showing the anteriorly positioned His bundle (C) and its lateral aspect (F). Distance between red dots- 0.6 mm, blue dots- 1.3 mm, green dots- 2.4 mm. AO- aorta, HB- His bundle, IVC- interventricular septum, LBB- left bundle branch, LV- Left ventricle, PB-penetrating bundle, RBB-right bundle branch. (Scale bar represents 0.5 mm).

The technique of micro-CT imaging of the heart was refined using the rat heart. Images of sections from rat hearts stained with a range of I_2_KI concentrations are shown in Fig. 1. For all concentrations tested, there was differential X-ray attenuation between contractile myocardium (high attenuation) and the CCS tissue (low attenuation), and an excellent morphological representation of the anatomical details of the heart including the valves, great vessels and muscular structure. We were able to identify the atrioventricular conduction axis including the penetrating bundle, His bundle, the bundle branches and the Purkinje network. The lowest I_2_KI concentration that allowed for high quality contrast enhancement was 3.75% with a incubation time of two days (Fig. 1C,G). It was possible to identify the CCS in a heart stained with 1.87% I_2_KI, but the ventricular endocardium was not stained homogeneously (Fig. 1D,H). To confirm the identity of the various structures, we attempted to make sections from frozen and paraffin-embedded blocks of the I_2_KI stained tissue, but found that, if I_2_KI was not removed after micro-CT scanning, the tissue was brittle and it was not possible to obtain satisfactory tissue sections. Therefore, the I_2_KI was leached from the hearts by immersion in PBFS for 2–5 weeks with weekly changes of PBFS. The time taken for complete leaching of I_2_KI was dependent on I_2_KI concentration, I_2_KI incubation time and specimen size, and ranged from two weeks for the lower concentrations to five weeks for the higher concentrations. Leaching was noted to be much quicker from dissected heart tissue, in which cut myocardial surfaces were exposed to the leaching solution. Fig. 2A,B shows two micro-CT images virtually sectioned from the micro-CT data to match Fig. 2D,E showing sections from wax-embedded blocks, stained with Masson’s trichrome - there is excellent correspondence and the images demonstrate that the penetrating bundle, His bundle and bundle branches are distinguishable from the surrounding structures in the micro-CT images. Differential attenuation is also observed between the surrounding connective tissue and contractile myocardium. By thresholding the differential attenuation between contractile myocardium and the CCS tissue, it was possible to ‘segment’ the CCS tissue, isolating it from the surrounding tissues, and generate a 3D representation of the CCS tissue. 3D images of the penetrating bundle and His bundle in a normal rat heart are shown in anterior and lateral orientations in Fig. 2C,F. In Fig. 2F, the penetrating bundle is seen (embedded within the central fibrous body), which connects the inferior nodal extension (not shown) to the His bundle and bundle branches anteriorly; the arching of the His bundle and bundle branches represents its position on the IVS.

Preparation of the rat hearts was such that collapsing of the atrial walls and blood residing in the chambers was unavoidable (Fig. 1), making identification of the SAN and Purkinje network difficult. The remainder of the results presented here are from rabbit hearts prepared with an improved method detailed in the methods section.

### 

#### Sinoatrial node of the rabbit heart

**Figure 3:**
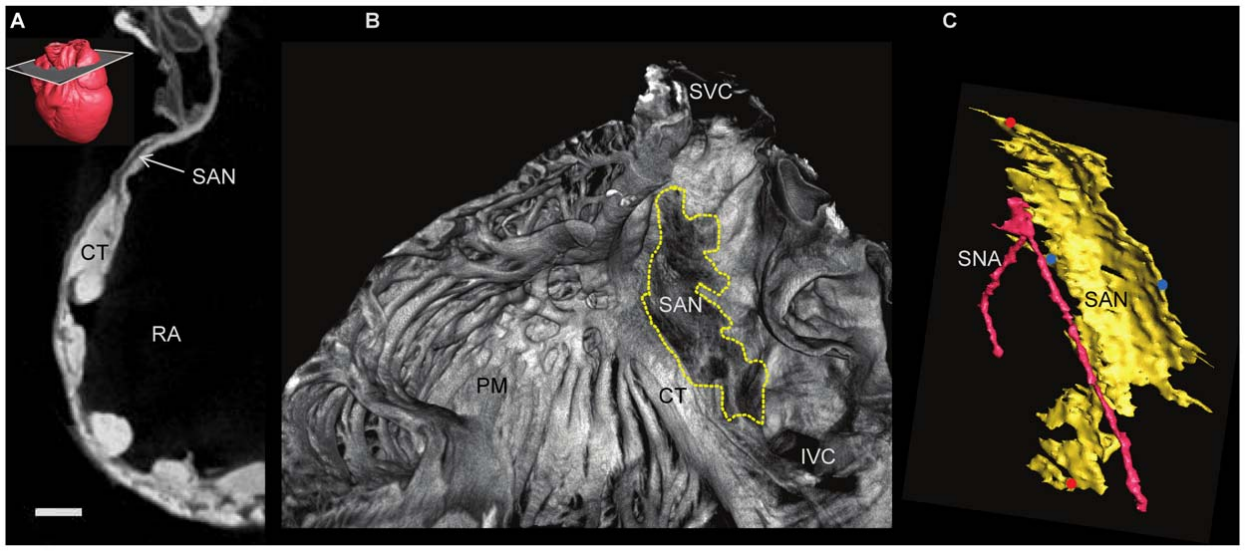
The atrioventricular conduction axis in rabbit. Longitudinal micro-CT images showing the penetrating bundle (A,D) and His bundle (B,E) in a rabbit heart stained with 7.5% I_2_KI for 3 days. Segmented 3D surfaces of atrioventricular conduction axis, showing the anteriorly positioned His bundle (C) and its lateral aspect (F). Distance between red dots- 1.1 mm, green dots- 7.8 mm. AOV- aortic valve, HB- His bundle, IVC- interventricular septum, LBB- left bundle branch, LV- left ventricle, PB- penetrating bundle, RBB- right bundle branch. (Scale bar represents 1 mm).

**Figure 4:**
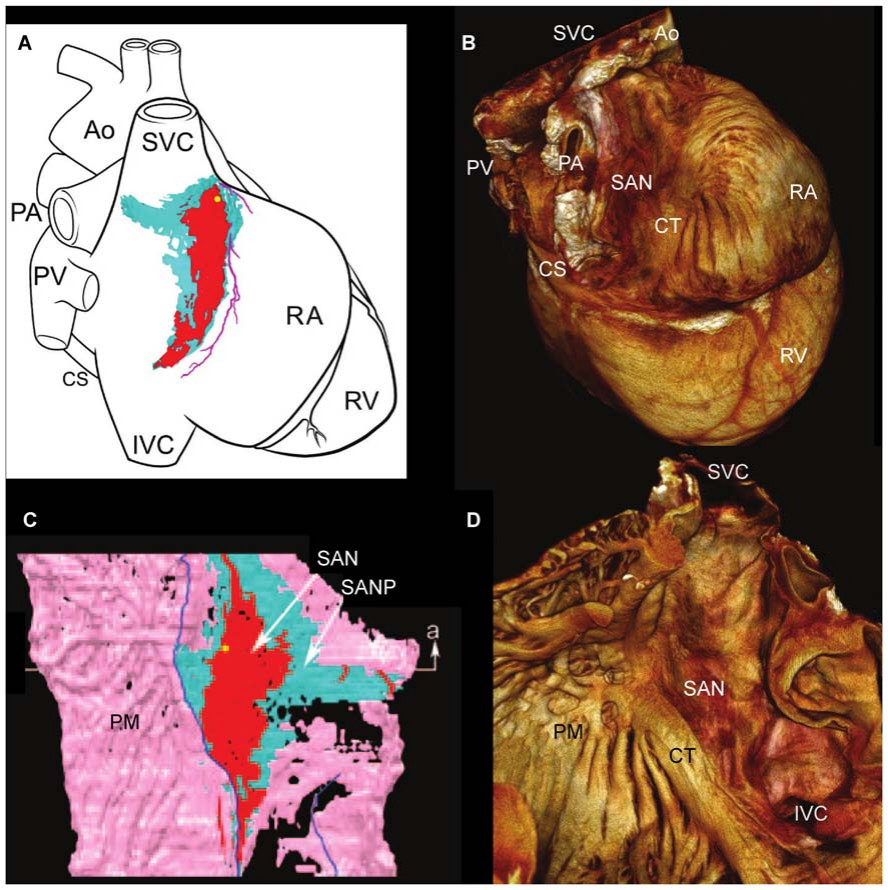
Identification of the sino atrial node. Transverse micro-CT image of the free wall of the right atrium showing the sino atrial node (A) in a rabbit heart stained with 7.5% I_2_KI for 3 days, with 3D rendering of the heart showing the associated plane of interest. Corresponding volume rendering (B) and Segmented 3D surface (C). Distance between red dots- 4.9 mm, blue dots- 2.5 mm. CT- crista terminalis, IVC- inferior vena cava, PM- pectinate muscle, RA- right atrium, SAN- sinoatrial node, SNA- sinus node artery, SVC- superior vena cava. (Scale bar represents 1 mm).

Analysis of the SAN confirms that it is not so much a localised compact node but an extended thin layer of conducting cells (Fig 3, Fig 4B,D). In the micro-CT images, the SAN was easily differentiated from the surrounding atrial myocardium as shown in Fig. 3A. It was a region of low attenuation (appears darker, Fig. 3A,B, Fig. 4B,D) in the intercaval region (between the superior and inferior venae cavae) in the rear wall of the right atrium. It extends along the lateral aspect of the crista terminalis, which demarcates the boundary between the smooth intercaval region and the pectinate muscles of the right atrial appendage (Fig. 3B, Fig. 4B,D). At the boundary of the intercaval region and the crista terminalis, there was evidence of interdigitation of the SAN and atrial muscle- the SAN tissue forks around the endocardial and epicardial surfaces of the crista terminalis (Fig. 3A). All of these are features of the rabbit SAN as identified by immunolabelling of marker proteins in physical sections [3]. Fig. 3B and Fig. 4D shows the extent of the rendered SAN as viewed from the endocardial surface of the right atrium. Fig. 3C shows the rendered SAN running alongside the sinus node artery. The volume of the rendered SAN was 1.01 mm^3^ in the rabbit heart shown. The attenuation data also contains evidence of a progressive increase in attenuation from the centre of the SAN towards its periphery and continuing to increase towards the surrounding atrial myocardium.

### Atrioventricular Node of the Rabbit Heart

**Figure 5:**
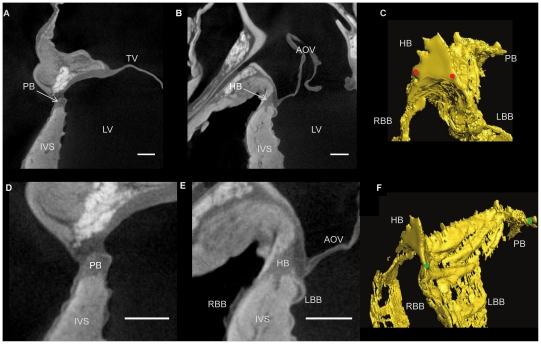
3-dimensional representation of the Purkinje Network. 3 rabbit hearts stained with 7.5% I_2_KI for 3 days (A-C Rabbit Heart 1) and 3.75% I_2_KI for 5 days (D-F Rabbit Heart 2, g-i Rabbit heart 3). Volume renderings viewed from base to apex (A,D,G), with corresponding segmented 3D surfaces in transverse (B,E,H) and longitudinal orientation (C,F,I). Volumes of segmented data- 4.2 mm^3^, 5.5 mm^3^, 15.8 mm^3^ respectively. Coloured dots represent Purkinje network maximum widths- distance between green dots- 13.5 mm, red dots- 17.1 mm, blue dots- 14.4 mm. IVS- interventricular septum, PM- papillary muscle.

**Figure 6:**
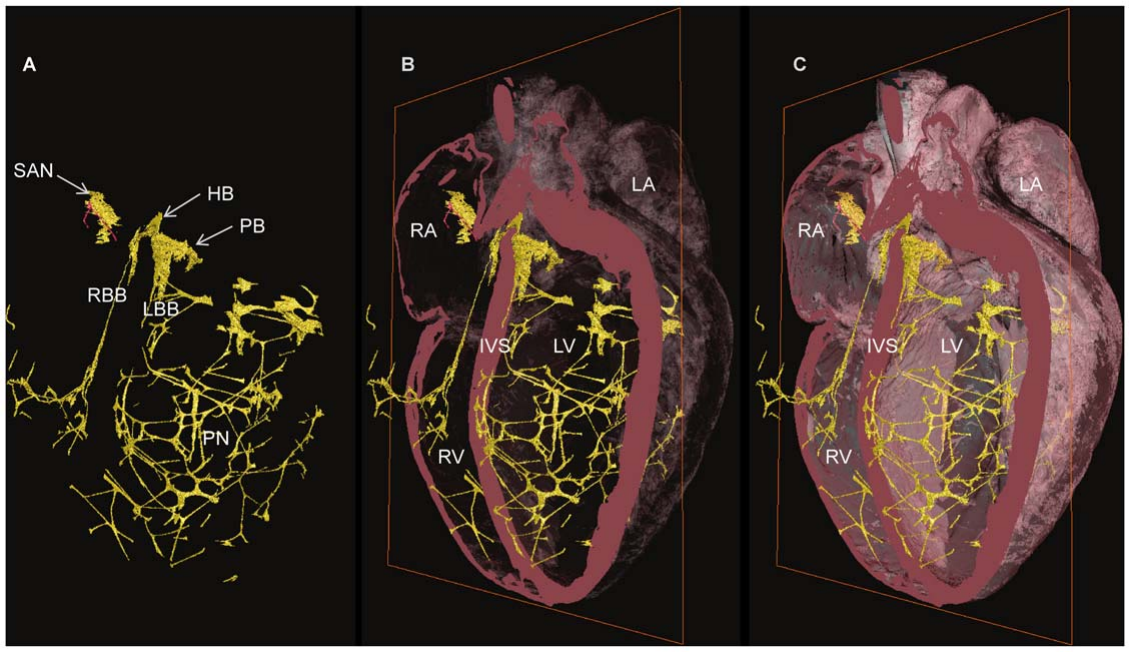
3-D representation of the CCS in failed rabbit heart. Segmented 3D surface showing the three major components of the CCS (yellow) overlaid with the longitudinally cropped cardiac mass (pink). Decreasing levels of cardiac mass transparency (A-C) show the true in situ position of the CSS. HB- His bundle, IVS- interventricular septum, LA- left atrium, LBB- left bundle branch, LV- left ventricle, PB- penetrating bundle, PN- Purkinje network, RA- right atrium, RBB- right bundle branch, RV- right ventricle, SAN- sinoatrial node.

**Figure 7:**
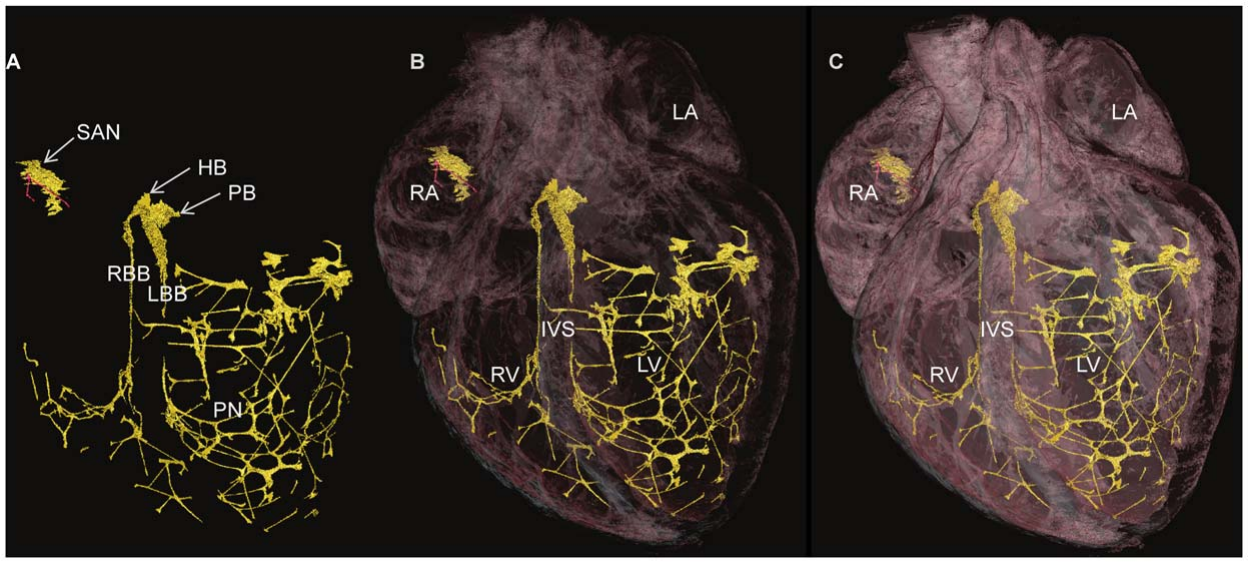
3-D representation of the CCS in failed rabbit heart. Segmented 3D surface from showing the three major components of the CCS (yellow) (A). Overlaid with cardiac mass (pink) at decreasing levels of transparency (B,C) showing the penetrating bundle emerging from the right atrium and transversing the IVS as the His bundle (in true in situ position). HB- His bundle, LA- left atrium, LBB- left bundle branch, LV- left ventricle, PB- penetrating bundle, PN- Purkinje network, RA- right atrium, RBB- right bundle branch, RV- right ventricle, SAN- sinoatrial node, IVS- interventricular septum.

**Figure 8:**
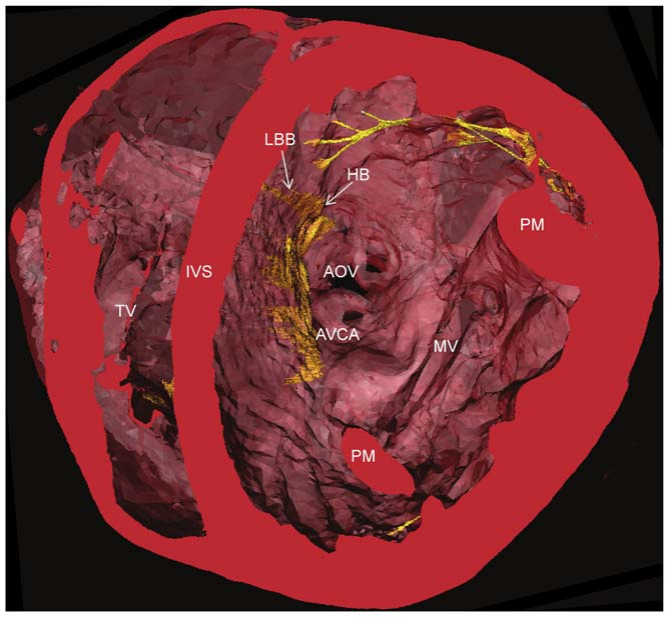
Trasnsverse course of the atrioventricular conduction axis. Rabbit heart segmented 3D surface, cropped transversely to show the relationship of the conducting tissue (yellow) with the surrounding cardiac mass (red) as it transverses the top of the interventricular septum. AOV- aortic valve, AVCA- atrioventricular conduction axis, HB- His bundle, IVS- interventricular septum, LBB- left bundle branch, MV- mitral valve, PM- papillary muscle, TV- tricuspid valve.

It was possible to identify the penetrating bundle, His bundle and bundle branches, along with the associated contractile myocardium and connective tissue (Fig. 5A,B,D,E). The 3D representation of the atrioventricular conduction axis in the rabbit (Fig. 5C,F) shows it to be similar to that in the rat (Fig. 2C,F). The arching of the structure represents its position on the IVS (Fig. 5C,F and Figs. 6,7). As the bundle branches are traced along the IVS it is evident that at their origin they appear as ribbon like structures (Fig. 5F), which become thinner and more ‘cord-like’ as they give branches to the purkinje network (Figs. 6,7). When viewed in a transverse section, the AV conduction axis can be seen to take origin in the expected location between the coronary sinus and the membranous septum at the apex of the triangle of Koch and can be followed around the circumference of the aortic valve annulus (Fig. 8).

### Purkinje System of the Rabbit Heart

**Figure 9:**
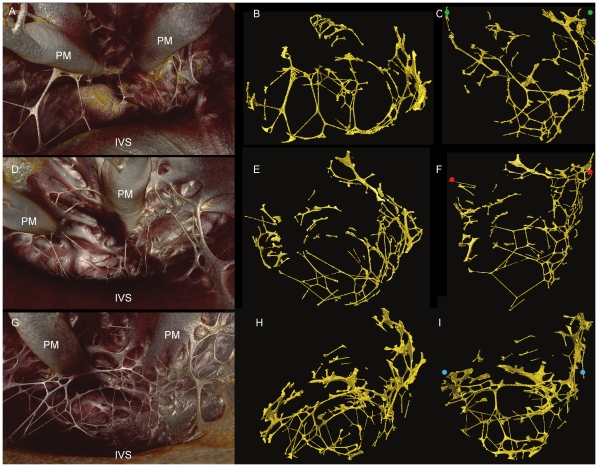
Comparison of existing and current models of the sino atrial node. Existing model of the SAN in rabbit (modified from Dobrzynski et al. 2005) constructed from immunohistochemically stained sections (A,C), superimposed onto a schematic representation of the heart showing the SAN epicardial position (A), and its relationship with the myocardium on the endocardial surface (C). Volume rendering of the current model in rabbit of the SAN, showing its true position on the epicardial (B) and endocardial (D) surfaces. Ao- Aorta, CS- coronary sinus, IVC- inferior vena cava, PA- pulmonary artery, PM- pectinate muscle, PV- pulmonary veins, RA- right atrium, SAN- sinoatrial node, SANP- sinoatrial node periphery, SVC- superior vena cava.

Free running Purkinje fibres are difficult to trace in any destructive sectioning technique because they are lost following sectioning, but in the micro-CT images they were easily identified, because in the imaged samples they were running in air. However, fibres running on the endocardial surface of the ventricles were more difficult to discriminate from the contractile myocardium. It was possible to segment some fibres running on the endocardial surface, because they appeared as protrusions on the surface of the myocardium continuous with the free-running fibres, but because these are small structures with a relatively small differential attenuation, this aspect of the segmentation was largely a manual process and unlikely to be complete. The ventricular Purkinje network is presented in three hearts from normal rabbits (Fig. 9). The least differentiated components of the His-Purkinje system are the right and left bundle branches (RBB, LBB) as they descend the IVS. This is because, in this area, the differential contrast between the (lower attenuating) conducting tissue and the (higher attenuating) working myocardium is smaller than in other areas of the CCS. indeed, in some parts of the bundle branches, the conducting tissue had a higher attenuation than the surrounding working myocardium.

In Figs. 6 and 7, the rendered His-Purkinje system is shown with its relationship to the surrounding myocardium. This shows that the LBB emerged as a ribbon-like structure (∼2 mm wide) that draped over the IVS at its widest point spanning ∼4.5 mm (e.g. Fig. 6, Fig. 5C,F). In contrast, the RBB emerged as a thin structure (∼0.75 mm wide), but still with a ribbon-like appearance (e.g. Fig. 7, Fig. 5C,F). The main branches can be traced down the IVS, with the LBB running vertically in the same plane as the His bundle; in contrast, the RBB projects anteriorly before taking a more vertical route down the IVS (Fig. 5F, Figs. 6,7). Figs. 6,7,9 show that the Purkinje network occupies the luminal cavity, with large portions of the network running from one side of the lumen to the other and, therefore, in life, running within the flowing blood. The data suggests that the Purkinje network in the left ventricle is more extensive than that in the right ventricle (Figs. 6,7). Finally, Fig. 9 shows that there is substantial variation in the His-Purkinje system between hearts. There are differences in fibre branching and thickness and in overall network volume (4.2 mm^3^, 5.5 mm^3^, 15.8 mm^3^; Fig. 9). However, there are similarities in terms of connections in all three samples (Fig. 9). For example, there are common branchings from the IVS to the ventricular free wall and from the IVS directly to the papillary muscles (Fig. 9).

### Variations of the CCS in Rat and Rabbit

Although the major components of the CCS are associated with the same anatomical landmarks in both rat and rabbit, there is evident variation in their morphology. The His bundle of the rat appears flattened and broader (Fig. 2) in comparison to the more narrow and elongated structure seen in rabbit (Fig. 5). The bundle branches and accompanying Purkinje networks are different in the two species; both bundle branches in the rat appear as uniform ribbon-like structure with widths of ∼0.5 mm (Fig. 2C,F), in rabbit the morphology of the LBB is quite different, the LBB emerges as a ribbon-like structure (∼2 mm wide) that drapes over the IVS, at its widest point spanning ∼4.5 mm (Fig. 5CF, Figs. 6,7) The RBB emerges as a thin structure (∼0.75 mm wide), but still has a ribbon like appearance (Fig. 5C,F, Figs. 6,7). The main branches can be traced down the IVS, with the LBB continuing distally in the same plane as the more proximal His bundle. In contrast the RBB projects anteriorly before taken a more vertical route down the IVS. The left ventricular Purkinje network in rat is localised to the apical region, creating a scoop-like appearence, with the majority of branches running on the endocardial surface towards the base with no cross luminal branching. In contrast the left ventricular Purkinje network of the rabbit appears as a web-like structure spanning the entire luminal cavity, with numerous branches traversing the cavity (Figs. 6,7,9).

### Summary

In Figs. 6 and 7, the whole of the rendered CCS (not just the Purkinje network) in one rabbit heart is shown superimposed on different views of the heart. This is the first time that such views have been shown for any species.

## Discussion

This study has shown that contrast enhanced micro-CT offers a non-destructive method to obtain a high-resolution map of the 3D disposition of the CCS within the heart. High resolution, non-invasive imaging of the 3D morphology of the CCS will have several important benefits. It will: a) improve the understanding of CCS anatomy and function; b) inform the development of ‘virtual hearts’- anatomically and biophysically-detailed mathematical models of the heart for education, research, planning surgical treatments and drug discovery; c) inform cardiac ablation procedures; d) provide better guidance for implantation of prosthetic aortic valves to avoid LBB block; and e) guide the planning of reconstructive surgery for congenital heart defects, to avoid damage to the CCS and reduce the need for pacemakers.

The figures presented here provide confirmation of many previous studies [2], [3], [4], [5], but are the first intrinsically 3D representation of the relationships between the conducting tissue and the cardiac anatomy, and the first to show the three major components of the CCS within datasets captured from single intact hearts.

### Comparison of Micro-CT to Other Techniques

During this study, hearts were carefully prepared by *in situ* perfusion to avoid the contrast degradation caused by the presence of luminal blood, and to provide uniform arterial fixation. During fixation, application of the contrast agent, and during preparation for scanning, care was taken to maintain the heart in an ‘inflated’ state. Without such care the atria are prone to collapse, and views from the inside of the atrial chambers are not possible. The micro-CT technique used in this study to identify and visualise the CCS has numerous advantages over current methods: (i) the technique is non-destructive and there is no distortion of the heart caused by dissection, embedding or freezing, which will be the case with serial sectioning (see Introduction). (ii) the staining by the contrast agent is reversible, so that samples are preserved for any future studies such as light microscopy. (iii) the technique achieves high resolution, which results in a more accurate representation of the CSS. In this study, the resolution was ∼18 µm. In studies involving serial sectioning, the resolution was 60–340 µm [3], [4]. To achieve a similar level of a resolution by a sectioning-based technique would require a high level of technical expertise and resources. Furthermore, to obtain such a set of sections from a small mammalian heart in its natural blood-filled disposition would require fixation and embedding in wax or plastic of an intact heart with atrial walls as thin as 0.2 mm. This would be a severe technical challenge. Although the resolution in this study is comparable to the resolution (∼25 µm) in a previous study involving high resolution MRI [10], micro-CT offers inherent isotropic resolutions while avoiding the issue of diminishing signal:noise ratio experienced with MRI at high resolutions. (iv) the technique is fast. In this study, imaging of one sample ranged between ∼20–50 minutes. In contrast, comparable serial sectioning work will take many days, and in the study of Li et al. MRI was stated to have taken many hours [10]. However, the micro-CT technique has a disadvantage: the ability of contrast enhanced micro-CT to discriminate between different tissues is limited compared to traditional sectioning and staining (although perhaps better than that of MRI).

Furthermore, existing techniques use small sample preparations removed from whole hearts by dissection, and therefore do not allow simple visualisation of the relationship between the CCS and the surrounding contractile myocardium. Resultant 3D renderings from such preparations are subsequently placed onto schematic diagrams of the whole heart subjectively [3], [4]. This potential source of error is not a limitation of the current study.

### Contrast Enhancement

The mode of contrast enhancement by iodine staining remains incompletely understood. One hypothesis is that the molecular iodine becomes immobilised within the structure of glycogen- the basis of simple tests for polysaccharides [22], [23]. While this provides a rational basis for the greater attenuation of myocardium than of fibrous tissue, it does not so obviously explain the lower attenuation that we have found generally for tissue of the CCS. The myocytes of the CCS are reported to have high glycogen content, and the classic histological stain known as Periodic Acid Schiff (PAS) is used to provide discrimination between the CCS and surrounding tissue [6], [7]. This apparent anomaly may be explained by the presence of glycogen-rich compartments in myocytes of the CCS, but a lower average density over the sectional area encompassed by a group of voxels. Myocyte density can be lower in the CCS (especially in parts of the sinoatrial and atrioventricular nodes) than in the tightly-packed contractile myocardium, and this may also contribute to the lower attenuation.

There is obvious potential for the current technique to be applied to the reconstruction of the CCS in larger hearts, including human hearts. Contrast enhancement of the CCS is achieved by the differential uptake of the contrast agent by different tissue types. In the present method contrast agent is taken up by the tissue through the process of diffusion therefore this may be a limitation when preparing larger samples because contrast may be lost in more superficial parts (too much iodine), before sufficient contrast is obtained in deeper parts of the tissue. It may therefore be necessary to develop a perfusion-based technique in which the iodine contrast is delivered via the arterial system.

### Segmentation of the CCS

The delineation of the complete CCS required use of various image analytical techniques. For the intra-ventricular Purkinje network, in which the structures run in air, an instantaneous volume rendering technique (VRT) can be used by windowing an opacity curve to include voxel values of interest and selecting a 3D colour map. The resultant 3D volume is rendered using a VRT render mode in which voxels are assigned a level of opacity (0–100%) calculated from their CT attenuation value. This complex procedure is versatile and time efficient and can easily combine characteristics of surface rendering and maximum intensity projection techniques, and offers improved definition of contours and semi-transparent displays over those techniques. For the SAN, a volume rendering that provided an image of the endocardial surface of the right atrium was most informative; use of VRT allowed an area to be identified that matched well with the landmarks of the crista terminalis and caval vein openings. However VRT does not allow visualisation of the AVN without digitally dissecting surrounding tissues, because the differential attenuation is variable, on account of the various tissue types that surround the AV conduction axis. In the samples presented here, the attenuation differences between the CCS and the surrounding myocardium were higher in the rat than in the rabbit. Although the iodine protocol for rabbit may benefit from further optimisation, this may represent a real difference between the species at the cellular level.

### Comparison to Previous Studies

The shape and volume of the rendered SAN of the rabbit heart in this study are similar to those reported by Dobrzynski et al. (2005) (Fig. 3,4), who used a more time consuming and labour intensive serial sectioning technique. Whereas the volume of the rendered SAN was 1.01 mm^3^ in this study, it was 1.4 mm^3^ in the study of Dobrzynski et al. (2005). Fig. 3B shows the SAN in the rabbit heart to be an extensive structure, unlike the ‘textbook view’ of the SAN, which is normally portrayed as a small nodule at the junction of the superior vena cava and the right atrium [1]. In fact the leading pacemaker site in the rabbit can be located at any site along the length of the crista terminalis from the superior to the inferior vena cava [1], and the extent of the SAN in the current study (Fig. 3B, Fig. 4B,D) is consistent with this.

Consistent with Tawara’s original histological description of the atrioventricular conduction axis [24], our 3D representation confirms that the atrioventricular conduction axis originates in the expected location between the coronary sinus and the membranous septum at the apex of the triangle of Koch and the axis can be followed around the circumference of the aortic valve annulus (Fig. 8). The 3D rendering of the atrioventricular conduction axis of the rabbit heart as shown in Fig. 5C,F is qualitatively and quantitatively similar to the 3D rendering of the atrioventricular conduction axis of the rabbit heart obtained from serial sectioning [4]. The 3D rendering in this study is superior in that it shows the connections to the bundle branches (whereas that from Li et al., 2008 does not). However, the 3D rendering in this study fails to show the various sub-compartments (inferior nodal extension, transitional zone, compact node, upper and lower tracts of the penetrating bundle) within the AVN that are identified by Li et al. (2008) by appropriate staining and immunolabelling of marker proteins. The His-Purkinje network identified in this study (e.g. Figs. 6,7) is qualitatively similar to that described in previous studies [11], [12], [24]. Whereas the Purkinje network identified in this study appears more complete than in the case of MRI [11], it is not as complete as the Purkinje network identified by immunolabelling [12], but it is revealed in its true 3D relationship to the intact cardiac chambers.

### Variations of the Ventricular Conduction System in the Rat and Rabbit

The His bundle of the rat is flattened and broader than the more narrow and elongated structure seen in the rabbit. The bundle branches and accompanying Purkinje networks are also different in the two species. Both bundle branches in the rat are uniform ribbon-like structures with a width of ∼0.5 mm (Fig. 2). In the rabbit, the LBB was a ribbon-like structure ∼2 mm wide that draped over the IVS, at its widest point spanning ∼4.5 mm (Fig. 5C,F). However, the RBB was a relatively thin ribbon-like structure ∼0.75 mm wide (Fig. 5C,F); this asymmetry between the bundle branches is consistent with the work of Atkinson et al. (2011). The left ventricular Purkinje network in the rat was localised to the apical region, creating a scoop-like appearance, with the majority of branches running on the endocardial surface towards the base with no cross luminal branching (data not shown) In contrast, the left ventricular Purkinje network in the rabbit was a web-like structure spanning the entire luminal cavity, with numerous branches traversing the cavity (Figs. 6,7,9). In addition there was clear variation between individuals (Fig. 9) in contrast to the conclusions of Atkinson et al. (2011).

### Conclusion

Here we present a technique by which the major regions of the CCS can be rapidly imaged non-invasively using reversible contrast enhancement for micro-CT in small mammalian hearts. The clear advantage over traditional methods of gross dissection, light microscopy and immunofluorescence staining for investigating the CCS is that high-resolution (µm) representations can be obtained with a non-destructive method while preserving 3D geometry and spatial relationships. There is a worldwide effort to develop a ‘virtual heart’, an anatomically- and biophysically-detailed model that can be used to describe normal function and predict dysfunction with pathology or malformation [25]. Although there are excellent models of the working myocardium [26], predictive models of the whole heart must include the CCS. Contrast-enhanced micro-CT offers an efficient method to generate an accurate anatomical model of the heart including the CCS, that can be incorporated into such models.
