# Correction: Contrast Enhanced Micro-Computed Tomography Resolves the 3-Dimensional Morphology of the Cardiac Conduction System in Mammalian Hearts

**DOI:** 10.1371/annotation/1baecd19-92b6-4683-b7d7-39c13a3f2e15

**Published:** 2012-04-23

**Authors:** Robert S. Stephenson, Mark R. Boyett, George Hart, Theodora Nikolaidou, Xue Cai, Antonio F. Corno, Nelson Alphonso, Nathan Jeffery, Jonathan C. Jarvis

There was an error in the placement of the legends for Figures 3, 4, 5, and 9. The correct legends are:

Figure 3. Identification of the sino atrial node.

Transverse micro-CT image of the free wall of the right atrium showing the sino atrial node (A) in a rabbit heart stained with 7.5% I_2_KI for 3 days, with 3D rendering of the heart showing the associated plane of interest. Corresponding volume rendering (B) and Segmented 3D surface (C). Distance between red dots- 4.9 mm, blue dots- 2.5 mm. CT- crista terminalis, IVC- inferior vena cava, PM- pectinate muscle, RA- right atrium, SAN- sinoatrial node, SNA- sinus node artery, SVC- superior vena cava. (Scale bar represents 1 mm).

Figure 4. Comparison of existing and current models of the sino atrial node.

Existing model of the SAN in rabbit (modified from Dobrzynski et al. 2005) constructed from immunohistochemically stained sections (A,C), superimposed onto a schematic representation of the heart showing the SAN epicardial position (A), and its relationship with the myocardium on the endocardial surface (C). Volume rendering of the current model in rabbit of the SAN, showing its true position on the epicardial (B) and endocardial (D) surfaces. Ao- Aorta, CS- coronary sinus, IVC- inferior vena cava, PA- pulmonary artery, PM- pectinate muscle, PV- pulmonary veins, RA- right atrium, SAN- sinoatrial node, SANP- sinoatrial node periphery, SVC- superior vena cava.

Figure 5. The atrioventricular conduction axis in rabbit.

Longitudinal micro-CT images showing the penetrating bundle (A,D) and His bundle (B,E) in a rabbit heart stained with 7.5% I_2_KI for 3 days. Segmented 3D surfaces of atrioventricular conduction axis, showing the anteriorly positioned His bundle (C) and its lateral aspect (F). Distance between red dots- 1.1 mm, green dots- 7.8 mm. AOV- aortic valve, HB- His bundle, IVC- interventricular septum, LBB- left bundle branch, LV- left ventricle, PB- penetrating bundle, RBB- right bundle branch. (Scale bar represents 1 mm).

Figure 9. 3-dimensional representation of the Purkinje Network.

3 rabbit hearts stained with 7.5% I_2_KI for 3 days (A-C Rabbit Heart 1) and 3.75% I_2_KI for 5 days (D-F Rabbit Heart 2, g-i Rabbit heart 3). Volume renderings viewed from base to apex (A,D,G), with corresponding segmented 3D surfaces in transverse (B,E,H) and longitudinal orientation (C,F,I). Volumes of segmented data- 4.2 mm^3^, 5.5 mm^3^, 15.8 mm^3^ respectively. Coloured dots represent Purkinje network maximum widths- distance between green dots- 13.5 mm, red dots- 17.1 mm, blue dots- 14.4 mm. IVS- interventricular septum, PM- papillary muscle. 

